# Supplementary material for: Seminoma and Embryonal Carcinoma Footprints Identified by Analysis of Integrated Genome-Wide Epigenetic and Expression Profiles of Germ Cell Cancer Cell Lines
Source: PLoS One. 2014 Jun 2;9(6):e98330. doi: 10.1371/journal.pone.0098330 (PMC4041891; doi:10.1371/journal.pone.0098330)
Supplement: File S1 — ZIP file containing DMRforPairs output for significant regions. Please start from the html files. (ZIP) [file pone.0098330.s008.zip › figures/35684.pdf]

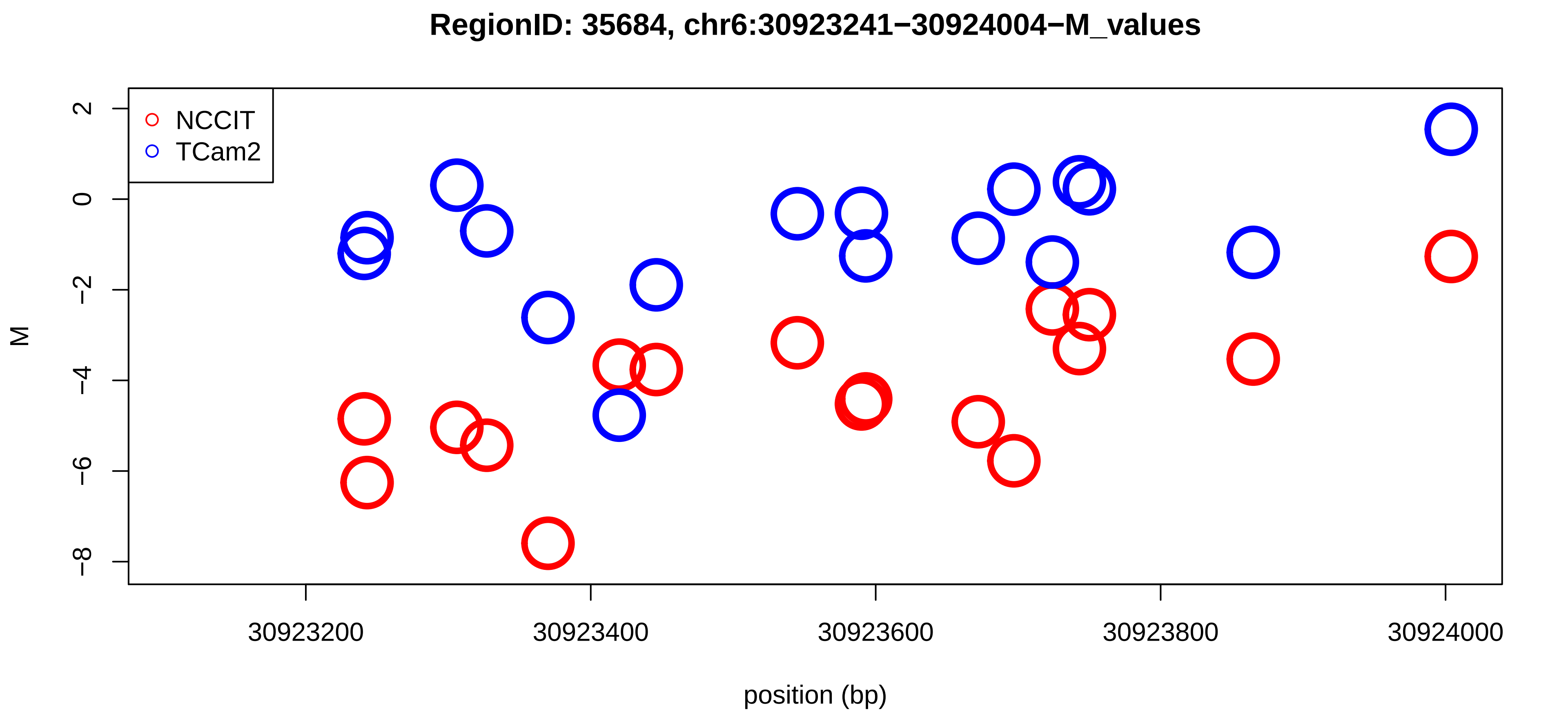
RegionID: 35684, chr6:30923241−30924004−M_values
NCCIT
TCam2
M
2
0
−2
−4
−6
−8
30923200
30923400
30923600
30923800
30924000
position (bp)

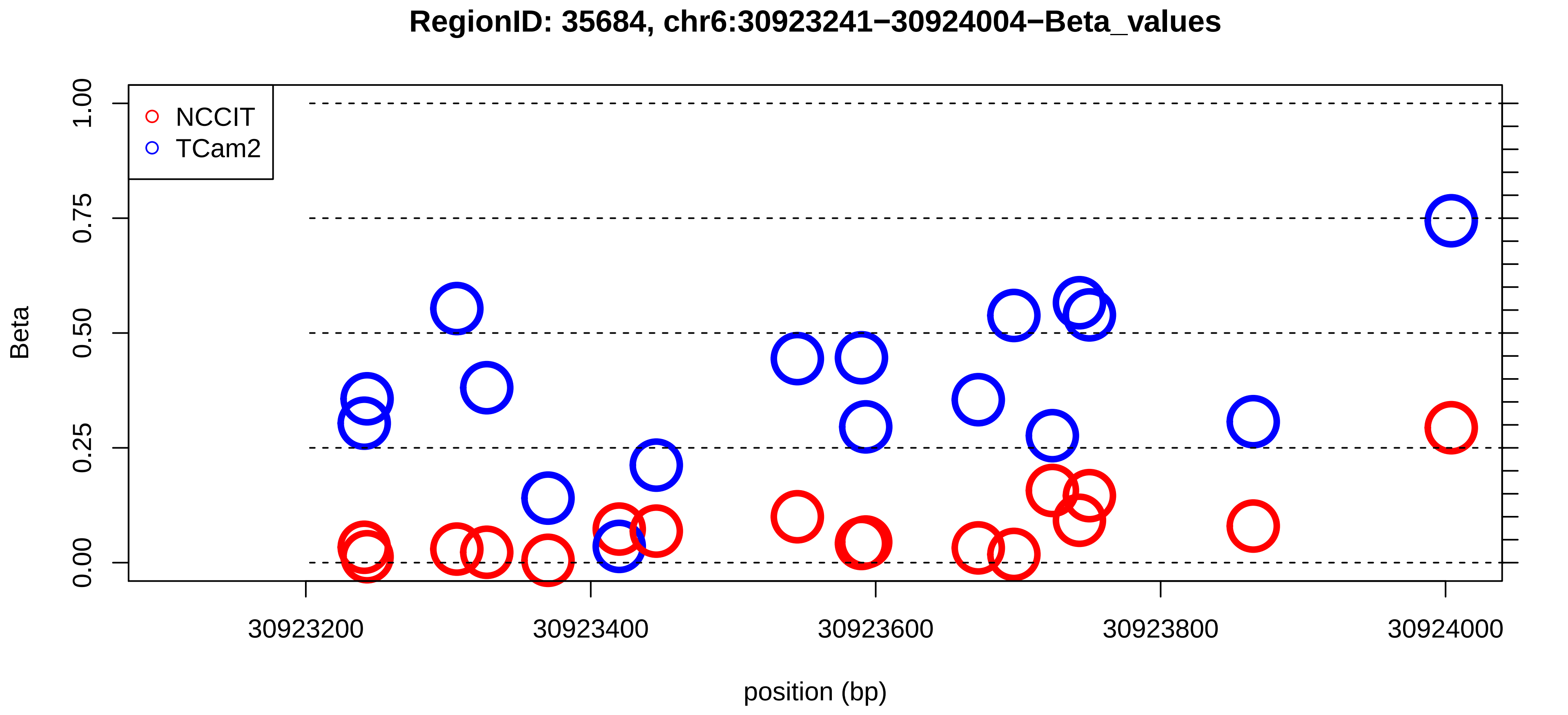
RegionID: 35684, chr6:30923241−30924004−Beta_values
NCCIT
TCam2
Beta
1.00
0.75
0.50
0.25
0.00
30923200
30923400
30923600
30923800
30924000
position (bp)

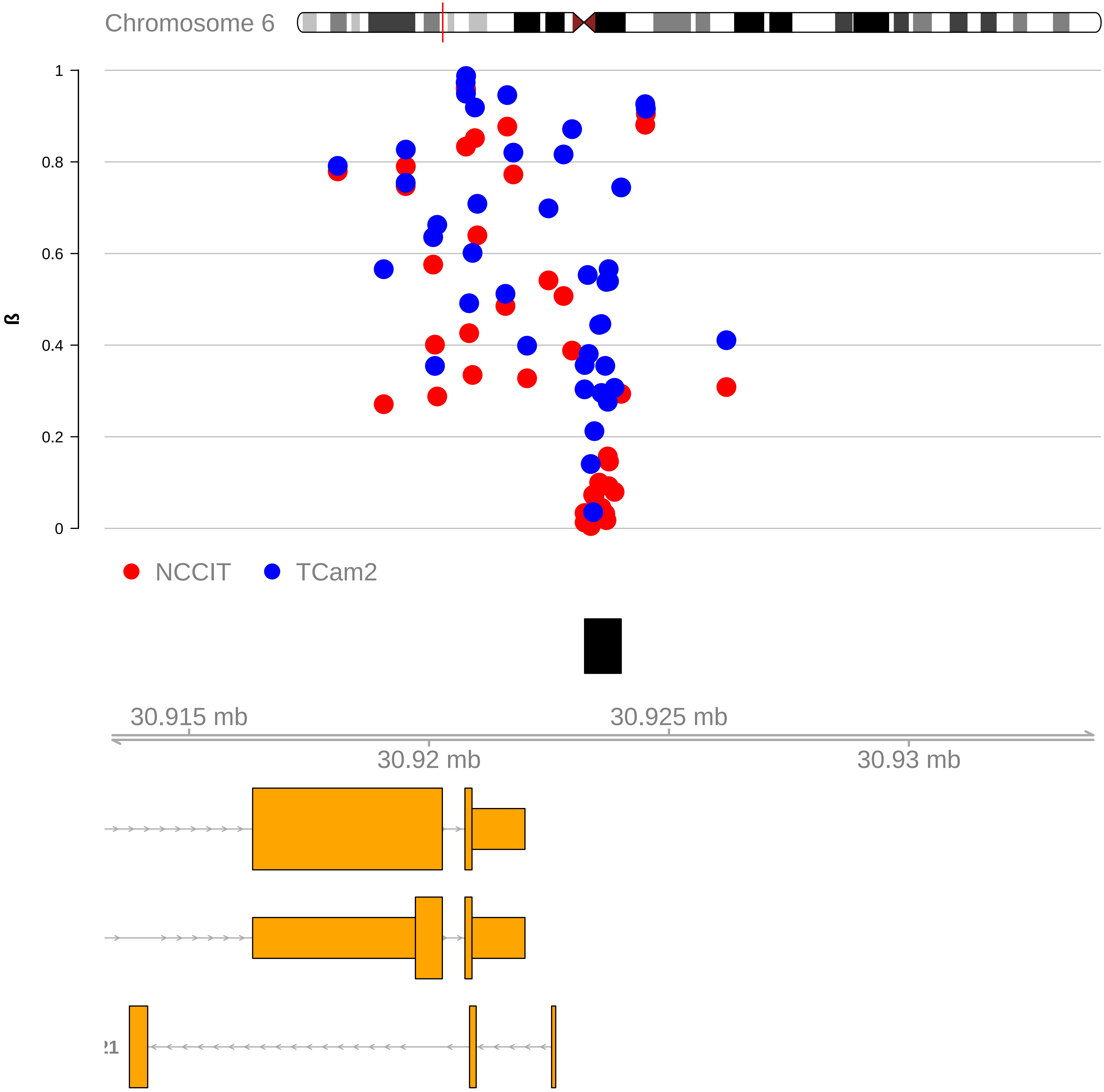

Chromosome 6
1
0.8
0.6
0.4
0.2
0
ß
NCCIT
TCam2
30.915 mb
30.925 mb
30.92 mb
30.93 mb
